# Black-white hole pattern: an investigation on the automated chronic neuropathic pain detection using EEG signals

**DOI:** 10.1007/s11571-024-10078-0

**Published:** 2024-02-28

**Authors:** Irem Tasci, Mehmet Baygin, Prabal Datta Barua, Abdul Hafeez-Baig, Sengul Dogan, Turker Tuncer, Ru-San Tan, U. Rajendra Acharya

**Affiliations:** 1 Department of Neurology, School of Medicine, Firat University, 23119 Elazig, Turkey; 2 Department of Computer Engineering, Faculty of Engineering and Architecture, Erzurum Technical University, Erzurum, Turkey; 3 School of Business (Information System), University of Southern Queensland, Toowoomba, QLD 4350 Australia; 4 School of Management and Enterprise, University of Southern Queensland, Toowoomba, QLD Australia; 5 Department of Digital Forensics Engineering, Technology Faculty, Firat University, Elazig, Turkey; 6 Department of Cardiology, National Heart Centre Singapore, Singapore, Singapore; 7 Duke-NUS Medical School, Singapore, Singapore; 8 School of Mathematics, Physics and Computing, University of Southern Queensland, Springfield, Australia

**Keywords:** Black-white hole pattern, EEG pain detection, Neuroscience, cortex map

## Abstract

Electroencephalography (EEG) signals provide information about the brain activities, this study bridges neuroscience and machine learning by introducing an astronomy-inspired feature extraction model. In this work, we developed a novel feature extraction function, black-white hole pattern (BWHPat) which dynamically selects the most suitable pattern from 14 options. We developed BWHPat in a four-phase feature engineering model, involving multileveled feature extraction, feature selection, classification, and cortex map generation. Textural and statistical features are extracted in the first phase, while tunable q-factor wavelet transform (TQWT) aids in multileveled feature extraction. The second phase employs iterative neighborhood component analysis (INCA) for feature selection, and the k-nearest neighbors (kNN) classifier is applied for classification, yielding channel-specific results. A new cortex map generation model highlights the most active channels using median and intersection functions. Our BWHPat-driven model consistently achieved over 99% classification accuracy across three scenarios using the publicly available EEG pain dataset. Furthermore, a semantic cortex map precisely identifies pain-affected brain regions. This study signifies the contribution to EEG signal classification and neuroscience. The BWHPat pattern establishes a unique link between astronomy and feature extraction, enhancing the understanding of brain activities.

## Introduction

Chronic neuropathic pain, a persistent affliction arising from nervous system damage or dysfunction, manifests as a consequence of abnormal neural processes (Sah et al. 2003). Neuropathic pain is often the result of another injury or pathological condition (Cohen and Mao 2014; Szczudlik et al. 2014). The chronicity of neuropathic pain, typically defined as enduring for a duration exceeding three months, imbues it with distinctive attributes that distinguish it from other forms of pain (Kim et al. 2023; Scholz et al. 2019). Notably, it primarily hinges on neural impairment rather than actual tissue damage or irritation within the body (Nishikawa and Nomoto 2017). This damage or dysfunction leads to impaired communication between nerve cells or the production of abnormal signals in neurons (Campbell and Meyer 2006). Etiological factors contributing to neuropathic pain encompass a spectrum of possibilities, including metabolic disorders like diabetic neuropathy, traumatic injuries, multiple sclerosis, infections, and more (Nudell et al. 2022; Sheldon et al. 2022). Furthermore, it is noteworthy that chronic pain can also manifest within neurological disorders whose etiology is not fully understood (Bonanni et al. 2022).

Chronic neuropathic pain significantly impacts one's quality of life (Mussigmann et al. 2022; Shinu et al. 2022). Unfortunately, conventional clinical drug therapies often fall short of mitigating its symptoms (Fisher and Clarkson 2023). Fundamentally, treatment strategies focus on pinpointing the root cause of the pain while alleviating its effects. Approaches encompass diverse methods including medication, neurostimulation, physical therapy, and psychotherapy, all aimed at curtailing and managing the pain (Vorobeychik et al. 2011). These techniques generally aim to improve the patient's quality of life, but it's important to note that the treatment journey demands sustained commitment and effort (Finnerup et al. 2018).

The diagnosis of clinical neuropathic pain necessitates a comprehensive evaluation by a healthcare professional, incorporating various diagnostic measures (Dansie and Turk 2013). A typical protocol to establish this diagnosis involves an in-depth patient history review, a thorough physical examination, electromyography, and specific laboratory tests (Lubec et al. 1999). Nevertheless, despite these meticulous steps, achieving a definitive diagnosis remains an intricate task. In this context, our objective is to employ automated diagnostic techniques utilizing EEG signals for chronic neuropathic pain. To fulfill this goal, we have devised a novel machine-learning approach capable of automatically classifying EEG signals obtained from individuals afflicted with chronic neuropathic pain.

### Literature review

The accurate diagnosis of pain type is paramount for tailoring effective treatment plans to patients (Finnerup et al. 2018). Nonetheless, precise pain diagnosis presents a formidable challenge. The literature presents numerous artificial intelligence-based approaches for pain classification, typically categorized into behavior-based and neurophysiology-based techniques (Cascella et al. 2023). In the literature, many AI-based methods have been proposed for different disciplines (Aydın 2022; Aydın and Onbaşı 2023; Özcelik and Altan 2023; Özçelik and Altan 2023). The literature review for the pain classification is presented below.

In a relevant investigation, Nezam et al. (Nezam et al. 2018) tackled the task of categorizing pain intensity into five distinct levels using EEG signals. To achieve this, they engaged in feature extraction within the alpha and delta bands of the EEG signal. Subsequently, they employed a sequential forward selection algorithm to identify the most pertinent features, followed by classification utilizing support vector machines (SVM). Their proposed method yielded an approximate classification success rate of 62%. It's essential to highlight that a key limitation of this research lies in the fact that pain induction was reliant on external stimuli. In their study, Chen et al. (Chen et al. 2022) harnessed the power of convolutional neural networks (CNN) to discern pain-related patterns within EEG signals. To this end, EEG data were meticulously acquired from a cohort of 10 individuals grappling with chronic back pain, followed by a rigorous classification task distinguishing between pain and no-pain states. This paper thoughtfully partitioned the EEG signals into 5-s segments with 4-s overlaps. Their proposed method yielded an impressive 83% area under the curve (AUC) value. However, it's prudent to acknowledge two salient limitations in this research: firstly, the reliance on signal segmentation with overlapping blocks, and secondly, the computational demands, characterized by high complexity. Anderson et al. (Anderson et al. 2021) examined EEG signals sourced from individuals afflicted with spinal cord injuries, employing the Higuchi fractal method for analysis. The study employed two distinct datasets, and the extracted features underwent classification via the Support Vector Machine (SVM) technique. Impressively, the proposed method achieved an accuracy rate of approximately 82%. In a related effort, Zolezzi et al. (Zolezzi et al. 2023a) conducted a comparative exploration of linear and nonlinear methods for classifying neuropathic pain. EEG signals, harvested from chronic neuropathic pain patients, were segregated into three groups based on pain severity and subjected to classification. The study partitioned signals into 1-min segments, with methodological outcomes evaluated against a statistical analysis framework. It's essential to acknowledge a significant limitation: the control group utilized a separate dataset, resulting in differences in the recording system. Elsayed et al. (Elsayed et al. 2020) ventured into pain level classification by applying a cold pressor test stimulator to 30 volunteer participants to elicit pain responses. EEG data were collected during this process, and four pain intensity categories (no pain/low/moderate/high) were distinguished. The classification phase employed Artificial Neural Networks (ANN), delivering a classification accuracy of 94.83%. Furthermore, the study unveiled a robust correlation between the alpha frequency band and pain intensity. However, it's imperative to note a key limitation in this study: the pain sensation was artificially induced via a stimulator.

As noted in the existing literature, neurophysiological-based pain classification methods predominantly rely on EEG-based approaches. Nevertheless, it's pertinent to acknowledge that many of these investigations utilize artificially induced pain through stimulators, with the EEG signals stemming from experimental setups rather than real patient scenarios.

### Literature gap

Based on the literature review, the identified gaps in the existing literature are as follows:

- While deep learning models dominate EEG signal classification, they tend to be computationally intensive.
- Dynamic pattern-based feature engineering models are scarce.
- A paper exists on EEG pain classification (Zolezzi et al. 2023a), but it did not provide detailed structural classification performance metrics.
- Most models primarily showcase their classification performance, with only a few offering explainable models that contribute to neuroscience.

### Motivation and our model

The inspiration behind introducing the astronomy-based pattern, BWHPat, in EEG signal classification stems from the pursuit of innovative methodologies in the detection and classification of brain disorders. The EEG signals are complex and often exhibit subtle patterns and variations. By drawing parallels between celestial phenomena and neural activities, we aim to leverage the unique characteristics of astronomical patterns to enhance feature extraction and classification within the realms of neuroscience and machine learning.

Astronomy, renowned for its ability to unravel intricate patterns in the vast expanse of space, offers a novel perspective for comprehending the complex patterns inherent in EEG signals. The introduction of BWHPat, modeled after the probabilistic patterns associated with black holes, serves as a bridge between the expansive patterns observed in the cosmos and the intricate neural patterns found in the human brain. This approach is driven by the belief that the dynamic and probabilistic nature of astronomical patterns can effectively address the intricacies and variations present in EEG signals.

The motivation for proposing BWHPat lies in its potential to provide a fresh and unconventional solution to the challenges in EEG signal classification. By incorporating astronomy-inspired concepts such as probability functions and dynamic graph generation, BWHPat aims to capture the richness of EEG data in a manner that aligns with the intricate patterns observed in celestial entities. The introduction of this model indicates our effort in proposing a novel feature extraction and classification techniques in the neuroscience field.

The BWHPat is an adaptive and dynamic selection model, allowing to respond effectively to the diverse patterns present in EEG signals. The motivation is to develop a robust and self-organizing features of BWHPat to achieve high classification accuracy.

One of our primary motivations is to address the gaps identified in the literature. The first gap highlights the dominance of deep learning models in EEG signal classification. Due to their differing strategies, traditional feature engineering models often struggle to achieve the same classification performance as deep learning models. While deep learning models employ dynamic weight detection through backpropagation-like techniques, Hinton's forward-forward (FF) model, as discussed in (Hinton 2022), offers a new approach. Hinton's research indicates that this model can be applied to feature engineering. As a result, we've become particularly interested in self-organized feature extraction models utilizing graphs. For our study, we employed the shape representation of the black-white hole. Through this shape, 14 patterns were formulated, leading to the proposal of a statistical method for optimal pattern selection. This strategy culminated in the conception of a self-organized dynamic pattern named BWHPat.

EEG pain classification is a fascinating research domain. While a paper on EEG pain detection exists, it primarily presents the dataset and outlines the statistical characteristics of the EEG signals. Our objective in this research is to present the classification outcomes of an EEG pain detection model.

Another crucial aim of this study is to produce explainable results. We've constructed a semantic cortex map based on the classification accuracy of the three examined cases. Using this cortex map, we've been able to provide interpretable insights regarding pain.

Our research introduces a novel feature engineering framework that yields results across all channels. This model comprises four phases: feature extraction, feature selection, classification, and cortex map creation. During the feature extraction phase, tools such as the tunable q-factor wavelet transform (TQWT) (Selesnick 2011), the proposed black-white hole pattern (BWHPat), and a statistical feature generator are utilized to derive multileveled textural and statistical features. The feature selection phase deploys iterative neighborhood component analysis (INCA) (Tuncer et al. 2020a) to pinpoint the most informative features. For classification, the k-nearest neighbors (kNN) (Peterson 2009) method is implemented to acquire channel-wise outcomes. Based on these results, we generated a semantic cortex map.

### Contributions and novelties

We introduce an innovative feature engineering model, and the distinct aspects and contributions of this research are outlined below:

*Novelties:*

- To the best of our knowledge, this paper presents the first EEG pain classification model.
- We are the pioneer in introducing an astronomy-based feature extraction model.
- We have elucidated chronic neuropathic pains using our proposed cortex map generation method.

*Contributions:*

- We have developed a new feature engineering methodology characterized by its self-organized feature extraction. This approach stands as a trailblazer in its domain. To substantiate the efficacy of our model, we introduced a feature extraction function termed BWHPat. Recognized as a self-organized feature extraction function, BWHPat facilitated the generation of textural features from the EEG signals we analyzed. Subsequently, we forged a novel feature engineering model leveraging BWHPat, particularly for EEG pain signal classification.
- To gauge the universal classification performance of our proposed BWHPat model, we tested it across three distinct cases. Remarkably, our model achieved classification accuracies exceeding 99% for all these scenarios. This accomplishment underscores the potency of our model in EEG signal classification, positioning it as a formidable contender against deep learning models. Thus, we offer a fresh alternative for signal classification, enriching the machine-learning community.
- Drawing from the insights garnered through our research, we crafted a semantic cortex map. This generated map unveils information regarding the brain's response to pain stimuli, marking a significant contribution to the realm of neuroscience.

## Dataset

The study employed a dataset collected from 36 chronic neuropathic pain patients (Zolezzi et al. 2023a, b). Their study (Zolezzi et al. 2023a, b) used a different EEG dataset to take control EEG signals, but their dataset has three classes. Therefore, we have used the original dataset. The demographics of the participants are as follows:

*Gender Distribution:* The patient pool consisted of 8 males and 28 females.

*Age Distribution:* The average age of the participants was 44 with a standard deviation of ± 13.98.

Questionnaires:

Participants completed two primary questionnaires to evaluate their pain levels and related conditions:

*Pain Detect Questionnaire (PDQ):* This tool is validated for the Spanish language and helps determine the neuropathic components of pain (Freynhagen et al. 2006).

*Brief Pain Inventory (BPI):* Also validated for Spanish, this inventory primarily focuses on pain severity and its interference with the patient's daily life (Erdemoglu and Koc 2013). Based on the BPI scores, patients were stratified into three pain severity categories:

*Low Pain:* Scores ranging from 0 to 3.

*Moderate Pain:* Scores ranging from 4 to 6.

*High Pain:* Scores ranging from 7 to 10.

The EEG data encapsulates recordings from 22 distinct channels, namely Fp1, Fp2, AFz, F7, F3, Fz, F4, F8, T7, C3, Cz, C4, T8, CPz, P7, P3, Pz, P4, P8, POz, O1, and O2.

The reference points for these recordings were the earlobes A1 and A2.

All EEG data was sampled at a frequency of 250 Hz, ensuring a high-resolution capture of the brain's electrical activity.

This dataset, encompassing EEG recordings, served as the foundation for our analyses and model evaluations in this study.

## The proposed black-white hole pattern

**Fig. 1:**
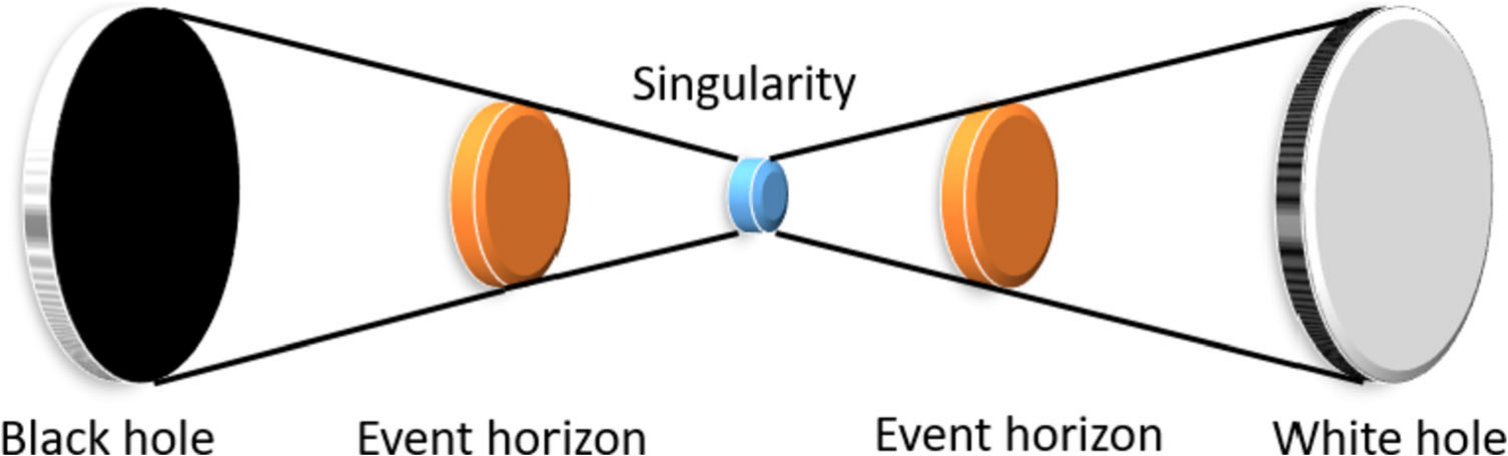
Graphical demonstration of the black-white hole

In feature engineering, conventional feature extraction functions have been employed as static patterns to generate features. However, these static patterns are limited in their ability to produce meaningful features from certain data blocks. Therefore, there is a need for a dynamic feature extractor to identify hidden patterns within the block. One of our innovations is the proposed BWHPat. In this section, our primary objective is to introduce a self-organized feature extraction function. While there are numerous graphs we could employ for this purpose, we aimed to tap into a popular research topic. Consequently, we utilized the shape of the black-white hole, which is depicted in Fig. 1.

**Fig. 2:**
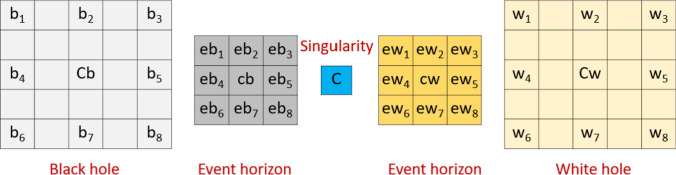
The matrices and values used to propose BWHPat. *b: values of the black hole, Cb: center of the black hole, eb: values of the event horizon of the black hole, cb: center of the event horizon of the black hole, C: singularity/center value, ew: values of the event horizon of the white hole, cw: center of the event horizon of the white hole

In this research, we developed a new pattern generator using the design inspired by the black-white hole sketch. We crafted a novel graph generator. For this modeling, we utilized five groups to represent the black hole, its event horizon, the singularity, the event horizon of the white hole, and the white hole itself. As a result, we employed two 5 × 5 matrices, two 3 × 3 matrices, and one 1 × 1 matrix. These matrices are illustrated in Fig. 2.

As depicted in Fig. 2, 69 values (calculated as 25 + 9 + 1 + 9 + 25) are required to execute this pattern generator. Using the pattern generator, we introduced a new self-organized feature extractor. We have outlined the model in the following steps to elucidate our proposal more clearly.

***S1:*** Create overlapping blocks for modeling and the length of each overlapping block is 69.

1 $$ \begin{array}{*{20}l}    {bl(h) = signal\left( {g + h - 1} \right),} \hfill  \\    {g \in \left\{ {{\text{1}},{\text{2}}, \ldots ,lng - 68} \right\},h \in \left\{ {{\text{1}},{\text{2}}, \ldots ,69} \right\}} \hfill  \\   \end{array}  $$

Herein, $bl$ means the block and $lng$ defines the length of the signal.

***S2:*** Generate the matrix shown in Fig. 2 using the overlapping block.

2 $$vb\left(j\right)=bl\left(j\right), j\in \left\{\mathrm{1,2},\dots ,25\right\}$$

3 $$veb\left(q\right)=bl\left(q+25\right), q\in \left\{\mathrm{1,2},\dots ,9\right\}$$

4 $$C=bl(35)$$

5 $$vew\left(q\right)=bl\left(q+35\right)$$

6 $$vw\left(j\right)=bl\left(j+44\right)$$

Herein, $vb$ defines the black hole vector, $veb$ means the event horizon of the black hole vector, $C$ represents the singularity value, $vew$ is the event horizon of the white hole vector and $vw$ defines the white hole vector. Here, we have generated the sub-blocks/vectors to generate matrices. The vector-to-matrix conversion is given below.

7 $$b\left(a,c\right)=vb\left(j\right), a\in \left\{\mathrm{1,2},\dots ,5\right\},b\in \left\{\mathrm{1,2},\dots ,5\right\}$$

8 $$w\left(a,c\right)=vw\left(j\right)$$

9 $$eb\left(z,n\right)=veb\left(q\right), z\in \left\{\mathrm{1,2},3\right\},n\in \left\{\mathrm{1,2},3\right\}$$

10 $$ew\left(z,n\right)=vew\left(q\right)$$

where $b$ defines the black hole, $eb$ means the event horizon of the black hole, $ew$ is the event horizon of the white hole and $w$ defines the white hole matrices.

***S3:*** Assign to values for feature extraction. (see Fig. 2).

***S4:*** Create the probable 14 patterns. By using these 14 patterns, 14 distinct binary features have been generated. The mathematical definitions of these binary feature extraction methods are given below.

The first four patterns are individual patterns. We have used four matrixes ($b,w,eb,ew$) to generate these binary feature extraction functions. Moreover, we have used the signum function as the main feature extraction function. We have defined the first four binary feature extraction functions below.

11 $$b{f}^{1}\left(r\right)=\rho \left({b}_{r},Cb\right),r\in \{\mathrm{1,2},\dots ,8\}$$

12 $$b{f}^{2}\left(r\right)=\rho ({w}_{r},Cw)$$

13 $$b{f}^{3}\left(r\right)=\rho ({eb}_{r},cb)$$

14 $$b{f}^{4}\left(r\right)=\rho ({ew}_{r},cw)$$

15 $$\rho \left({b}_{r},Cb\right)=\left\{\begin{array}{c}0,{b}_{r}<Cb\\ 1,{b}_{r}\ge Cb\end{array}\right.$$

Herein, $bf$ defines the binary features and $\rho (.,.)$ function is a signum function and we have utilized it as a main kernel function to generate binary features.

The 5th–8th patterns have been generated using the singularity value.

16 $$b{f}^{5}\left(r\right)=\rho \left({b}_{r},C\right)$$

17 $$b{f}^{6}\left(r\right)=\rho ({w}_{r},C)$$

18 $$b{f}^{7}\left(r\right)=\rho ({eb}_{r},C)$$

19 $$b{f}^{8}\left(r\right)=\rho ({ew}_{r},C)$$

The 9th pattern is created using the black hole and white hole.

20 $$b{f}^{9}\left(r\right)=\rho ({b}_{r},{w}_{r})$$

The 10th pattern has been created using event horizons.

21 $$b{f}^{10}\left(r\right)=\rho ({eb}_{r},{ew}_{r})$$

The 11th–14th patterns (bit groups) have been generated using holes and event horizons.

22 $$b{f}^{11}\left(r\right)=\rho ({b}_{r},e{b}_{r})$$

23 $$b{f}^{12}\left(r\right)=\rho ({w}_{r},e{w}_{r})$$

24 $$b{f}^{13}\left(r\right)=\rho ({b}_{r},e{w}_{r})$$

25 $$b{f}^{14}\left(r\right)=\rho ({w}_{r},e{b}_{r})$$

In this step, we have defined the used 14 patterns. However, there is the best suitable pattern selection problem. The solution to this problem is explained in Step 5 (see S5).

***S5:*** Select the best pattern using the Euclidean distance-based statistical moment. To implement this pattern selection function, we have created groups. The generated groups are shown below.

26 $$ \begin{array}{*{20}l}    {G^{1}  = b,G^{2}  = w,} \hfill  \\    {G^{3}  = eb,G^{4}  = ew} \hfill  \\   \end{array}  $$

The first four groups ($G$) are the individual groups. The remainder 10 groups are the combination-based groups and these groups are defined below.

27 $$ \begin{array}{*{20}l}    {G^{5}  = \varpi \left( {b,C} \right),G^{6}  = \varpi \left( {w,C} \right),G^{7}  = \varpi (eb,C),G^{8}  = \varpi (ew,C),} \hfill  \\    {G^{9}  = \varpi \left( {b,w} \right),G^{{10}}  = \varpi \left( {eb,ew} \right),G^{{11}}  = \varpi \left( {b,eb} \right),G^{{12}}  = \varpi \left( {w,ew} \right),} \hfill  \\    {G^{{13}}  = \varpi (b,ew),G^{{14}}  = \varpi (w,eb)} \hfill  \\   \end{array}  $$

where, we have used concatenation function ($\varpi (.,.)$) function to create these 10 groups. The used pattern selection function is defined below.

Firstly, we have calculated the general feature of the signal by using the below equation.

28 $$\mu \left(signal\right)=\frac{\sum_{t=1}^{lng}signal(t)}{lng}$$

29 $$\sigma \left(signal\right)=\sqrt{\frac{\sum_{t=1}^{lng}{\left(signal\left(t\right)-\mu \left(signal\right)\right)}^{2}}{lng-1}}$$

Herein, $\mu (.)$ function represents the average value calculation function and $\sigma (.)$ implies the standard deviation function. Herein, our objective is to compute the general statistical attribute of the used input. By using the Euclidean distance of the generated statistics, we have selected the best feature pattern.

30 $$D\left(e\right)=\sqrt{{\left(\mu \left(signal\right)-\mu \left({G}_{e}\right)\right)}^{2}+ {\left(\sigma \left(signal\right)-\sigma \left({G}_{e}\right)\right)}^{2}} ,e\in \{\mathrm{1,2},\dots ,14\}$$

31 $$idt={\text{min}}(D)$$

32 $$BF=b{f}^{idt}$$

where $D$ defines distances from the input signal and the created groups, $idt$ represents the index value of the minimum distance and $BF$ means of the final binary features. By using the selected binary features, we have generated map values.

***S6:*** Generate map value using the selected binary features.

33 $$mval=\sum_{z=1}^{8}BF(z)\times {2}^{8-z}$$

Here, $mval$ is the generated map value by using the selected binary features.

***S7:*** Repeat S1–S6 until the number of the blocks and generate a map signal.

***S8:*** Extract histogram of the map signal to generate feature vector.

34 $$feat=\theta (map)$$

where $feat$ is the feature vector with a length of 256 since we have coded map signal ($map$) with eight bits and $\theta (.)$ represents the histogram extraction function.

The proposed BWHPat has defined the above eight steps and this feature extractor is a self-organized feature extraction function since this extractor selects the most suitable pattern per the structure of the used data.

## EEG classification model

Our main goal is to examine the classification capabilities of the proposed BWHPat. To this end, we introduced a new EEG classification model incorporating a hybrid and multileveled feature extraction method. This method integrates TQWT (Selesnick 2011), statistical features, and the newly proposed BWHPat. During the feature selection phase, we employed INCA (Tuncer et al. 2020a), while kNN (Peterson 2009) was responsible for classifying the selected features for each channel. Using the classification outcomes from these channels, we formulated a semantic cortex map based on the achieved classification performance and a novel intersection model.

**Fig. 3:**
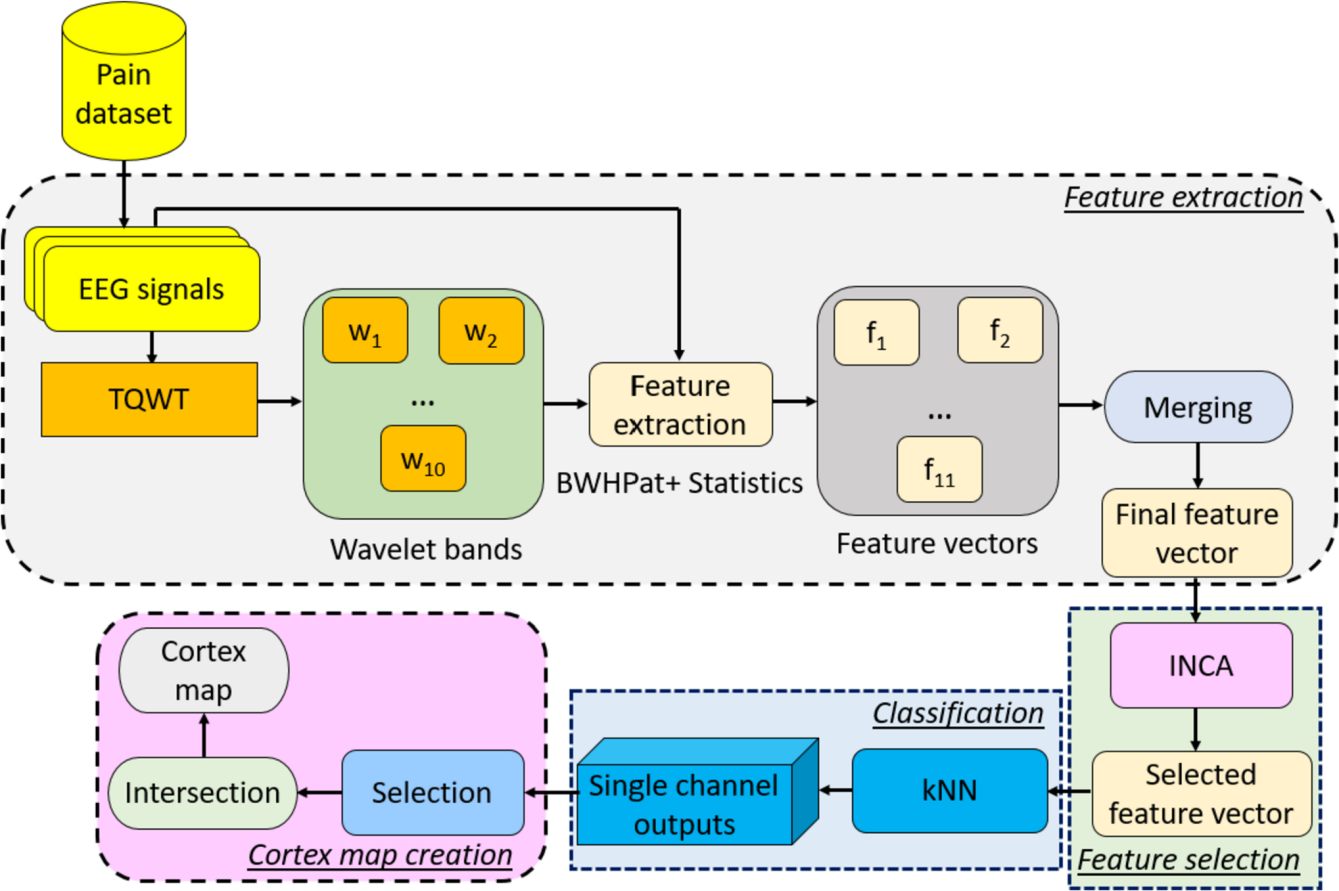
Schematic diagram of the proposed model. ** w: Wavelet bands (these bands, which are wavelet coefficients, have been generated by the TQWT, and they serve as wavelet filters). f: Individual feature vector

The visual representation of our proposed model can be seen in Fig. 3.

It can be noted from Fig. 3 that, the proposed feature engineering model contains four phases, and these phases are (i) feature extraction, (ii) feature selection, (iii) classification, and (iv) cortex map creation.

We generated features using both a statistical feature generator and BWHPat-based feature extractor, resulting in 11 inputs (comprising the raw EEG signal and 10 wavelet bands). The proposed hybrid feature extractor generates 270 features from each input, which includes 256 features from the proposed BWHPat and an additional 14 features extracted by the statistical feature generator. Consequently, 11 individual feature vectors ($f$) are generated and concatenated, resulting in feature vector with a length of 2970 (equal to 270 × 11). Subsequently, the INCA feature selector is used to select the most informative features from the generated 2970 features. Finally, the kNN classifier is employed to generate classifier-specific outcomes, with kNN providing channel-specific results. Information fusion techniques are then applied to obtain the optimal result.

To clarify the proposed model better, we have given more details about its phases below. Moreover, we have explained this model step-by-step.

### Feature extraction

The initial phase of our proposed model centers on feature extraction. We employed TQWT to derive wavelet bands in this phase, thereby establishing a multileveled feature extraction function (Selesnick 2011). We integrated two feature extraction functions: (i) the introduced BWHPat and (ii) a statistical feature extractor. While the statistical feature extraction function yields statistical features, our BWHPat is designed to produce textural features. Additionally, by harnessing wavelet bands, we extracted features specific to frequency bands and generated space domain features directly from the raw EEG signal. The steps for the feature extraction function we introduced are as follows:

***Step 1:*** Generate the wavelet bands deploying TQWT.

35 $$W=\psi (signal,\mathrm{1,3},9)$$

Herein, $W$ defines the wavelet bands, $\psi ()$ function represents the TQWT transformation, and we have used 1,3 and 9 values as the q-factor, redundancy, and number of levels, respectively. We have generated 10 (= number of levels + 1) wavelet bands using this wavelet transform.

The TQWT is chosen for frequency band extraction in the EEG pain classification model due to its following advantages. Firstly, wavelets perform well in multiresolution analysis as they can effectively capture both high and low-frequency components in EEG signals (Adeli et al. 2003). This capacity is crucial for comprehending the intricate dynamics of neural signals associated with pain perception. Additionally, wavelets offer exceptional time–frequency localization, enabling them to capture transient changes in EEG signals over time. This feature distinguishes them from traditional Fourier transform-based methods and renders them well-suited for analyzing non-stationary signals commonly encountered in pain-related brain activity. The wavelets can dynamically adjust their resolution and bandwidth based on the signal's characteristics. This adaptability is essential when working with EEG data, where neural signals exhibit considerable variability in terms of frequency and amplitude during different pain-related events. Finally, wavelet-based approaches provide an extensive set of features that can be extracted from EEG signals, facilitating the capture of unique patterns and characteristics associated with pain perception. Due to these advantages, we have chosen wavelets for feature extraction in the BWHPat model.

***Step 2:*** Extract the feature from the raw EEG signal and generate wavelet bands by deploying the proposed BWHPat and statistical feature extractor. The proposed BWHPat extract 256 textural features and the used statistical feature generator extracts 14 statistical features since the statistical feature generator uses 14 statistical moments and these moments are (1) Tsallis Entropy, (2) Shannon Entropy, (3) Renyi Entropy, (4) Sure Entropy, (5) Log Entropy, (6) Energy, (7) Higuchi, (8) Standard Deviation, (9) Variance, (10) Range, (11) Mean, (12) Median, (13) Minimum and (14) Maximum (Tuncer et al. 2020b). Using the generated wavelet bands, raw EEG signal and the used feature extraction functions, we have generated 11 feature vectors, and the length of each feature vector is equal to 270 (= 256 + 14).

36 $${f}_{1}=\varpi \left(\beta \left(signal\right),\xi \left(signal\right)\right)$$

37 $${f}_{s+1}=\varpi \left(\beta \left({W}_{s}\right),\xi \left({W}_{s}\right)\right), s\in \{\mathrm{1,2},\dots ,10\}$$

where $f$ defines the feature vector, $\beta ()$ is the proposed BWHPat feature extractor and $\xi ()$ means the statistical feature extraction function.

***Step 3:*** Merge the generated feature vectors to generate the ultimate feature vector.

38 $$X=\varpi \left({f}_{1},{f}_{2},\dots ,{f}_{11}\right)$$

Herein, the ultimate feature vector is denoted using $X,$ and the length of this feature vector is equal to 2970 (= 270 × 11).

***Step 4:*** Repeat Steps 1–3 until the number of the EEG segments and create a feature matrix.

### Feature selection

In the feature selection phase, we employed the Iterative neighborhood component analysis (INCA) (Tuncer et al. 2020a). We aimed to extract the most informative features from 2970 features. INCA is an enhanced iteration of the NCA feature selector, integrating additional functionalities for a more refined feature selection process (Goldberger et al. 2004).

It operates through an iterative mechanism, introducing a structured process that includes the generation of loss values. This iterative nature, coupled with the incorporation of a check-balance structure helps to distinguish INCA from NCA. The check-balance structure entails evaluating and balancing the contributions of various features, ensuring a comprehensive assessment of the feature space. This is particularly crucial for the INCA, as it employs a loss value generation function to calculate the loss values for all selected feature vectors. This process is crucial in determining the optimal selected feature vector, as it systematically analyzes the impact and significance of each feature within the dataset. Through this meticulous evaluation, the INCA feature selector identifies and prioritizes the most relevant features, contributing to the overall effectiveness of the feature selection process.

One of the key strengths of INCA is its ability to navigate through the feature space dynamically, adapting to the evolving characteristics of the dataset. The iteration process and the check-balance structure work synergistically to determine the optimal feature vector from the pool of selected feature vectors. This optimization is guided by the minimization of loss values, enhancing the selector's capability to identify the most discriminative features relevant to the classification task.

***Step 5:*** Generate the qualified indexes of the features by deploying the NCA feature selector.

39 $$index=\mathcal{N}\left(X,y\right)$$

where $index$ defines the qualified indices of the features, $\mathcal{N}(.,.)$ represents the NCA function and $y$ means the actual output.

***Step 6:*** Select feature vectors iteratively and compute the loss values of these feature vectors by deploying the kNN classifier.

40 $$ \begin{array}{*{20}l}    {sft^{{x - stv + 1}} \left( {dm,i} \right) = X\left( {dm,index\left( i \right)} \right),x \in \left\{ {stv,stv + 1, \ldots ,fnv} \right\},} \hfill  \\    {i \in \left\{ {{\text{1}},{\text{2}}, \ldots ,x} \right\},dm \in \{ {\text{1}},{\text{2}}, \ldots ,NoE\} } \hfill  \\   \end{array}  $$

41 $$loss\left(x-stv+1\right)=kNN\left(sf{t}^{x-stv+1},y\right)$$

Herein, $sft$ defines the selected feature vector, $stv$ implies the start value of the iteration, $fnv$ is the final value of the iteration, $loss$ means of the misclassification value array and $NoE$ represents the number of EEG signal.

***Step 7:*** Choose the best feature vector according to the computed loss values.

42 $$idx={\text{min}}(loss)$$

43 $$sfeat=sf{t}^{idx+stv-1}$$

where $idx$ defines the index of the minimum misclassification value and $sfeat$ is the ultimately selected feature vector.

### Classification

During the classification phase, we utilized the kNN (Peterson 2009) classifier to produce results. The kNN classifier is among the most widely used distance-based classifiers in literature. Furthermore, NCA can be viewed as the feature selection counterpart of kNN. Consequently, we combined INCA and kNN to achieve superior classification performance. The classification steps for the introduced BWHPat are detailed below.

***Step 8:*** Classify the selected feature vector by deploying the kNN classifier and generate the outcome.

44 $$out=kNN\left(sfeat,y\right)$$

Herein, $out$ is the outcome of the used channel.

***Step 9:*** Repeat Steps 1–8 until the number of channels and compute the classification results of each channel.

### Cortex map creation

The mathematical model used to produce interpretable results utilizing the cortex is presented in this phase. In this stage, we obtained channel-wise outcomes to pinpoint the active channels for the pain classification phase. The cortex maps are based on the amplitudes or types (alpha, beta, gamma, or theta) of the EEG signals. In this work, we developed the cortex map utilizing classification results. The step-by-step explanation of cortex map creation is given below.

***Step 10:*** Compute the classification accuracy for all channels using the outcomes generated during the classification phase for all three cases used.

45 $$ca{c}_{j}^{t}=\varphi \left(ou{t}_{j},y\right), j\in \left\{\mathrm{1,2},\dots ,24\right\}, t\in \left\{\mathrm{1,2},3\right\}$$

Here, $cac$ means of the classification accuracy and $\varphi ()$ is the classification accuracy calculation function. We have used three cases. Therefore, we have computed the classification accuracies for all cases.

***Step 11:*** Calculate the median values of the classification accuracies for all three cases used.

46 $$medval(t)=median\left(ca{c}^{t}\right)$$

where $medval$ defines the median value and we have computed three median values in this step.

***Step 12:*** Find the channels with classification accuracies that are higher than median values and store these channels as meaningful channels.

47 $$find\left(ca{c}^{t},medval\right)=\left\{\begin{array}{c}mc{h}^{t}\left(q\right)=j \wedge q=q+1, ca{c}_{j}^{t}>medval(t) \\ skip, ca{c}_{j}^{t}\le medval(t)\end{array}\right.$$

where $find(.,.)$ represents the finding function and $mc{h}^{t}$ is the meaningful channel of the t^th^ case.

***Step 13:*** Apply intersection operation to meaningful channels to get ultimate/general meaningful channels for pain detection.

48 $$umc=\bigcap_{t=1}^{3}mc{h}^{t}$$

Herein $umc$ is the ultimate/general meaningful channel for the pain classification using EEG signals.

Using the generated channels, we have presented interpretable results in the field of neuroscience and identified the most meaningful channels for addressing this problem.

Regarding the significance of the median and intersection functions in generating the cortex map, these functions play a crucial role in streamlining the identification of meaningful channels. The median values capture the central tendency of classification accuracies across different cases. Channels surpassing these median values provide a refined selection based on consistent performance.

The intersection operation further refines this selection by identifying channels that consistently exhibit meaningful classification across all three cases. The resulting ultimate/general meaningful channel ($umc$) represents a consolidated set of channels that collectively contribute to effective pain detection in EEG signals. This approach enhances the interpretability of the cortex map, offering insights into the specific brain regions affected by pain.

The 13 steps above have been defined in the proposed EEG signal classification model.

## Experimental results

In this section, the classification performances of the proposed feature engineering model have been presented.

### Experimental setting

A simple configured personal computer (PC) was used to implement this model, and this PC has 32 GB main memory, a 3.6 GHz intel processing unit, and a graphic card with 240 tensor cores. MATLAB (2023a) programming environment was used to implement the presented BWHPat model. Moreover, we implemented this proposal using CPU mode since our model is a lightweight EEG signal classification model. The presented EEG signal classification model used many methods and these methods are (i) TQWT (Selesnick 2011), (ii) the recommended BWHPat, (iii) statistical feature extractor (Tuncer et al. 2020b), (iv) INCA (Tuncer et al. 2020a), (v) kNN (Peterson 2009) and (vi) cortex map generator. To better define the presented model, we have listed the parameters of the used methods below.

*TQWT:*

- Q-factor (Q): 1, redundancy (r): 3, number of levels (J): 9.

*BWHPat:*

- The length of the used overlapping block: 69,
- Number of matrices: 5,
- Kernel: Signum,
- Number of patterns: 14,
- Pattern selection function: Euclidean distance-based statistical moment,
- The length of the features: 256,
- Type of the features: textural.

*Statistical feature extractor:*

- 14 statistical moments (= 7 linear + 7 nonlinear),
- Type of the features: statistical.

*INCA:*

- Solver of NCA: Stochastic gradient descent,
- A number of iterations of the NCA: Half of the number of EEG signals.
- Classifier: kNN with tenfold CV,
- Range of iteration: from 50 to 500,
- The number of selected feature vectors generated: 451 (= 500–50 + 1),
- Selection function: Feature vector with minimum misclassification rate.

*kNN:*

- k: 1, distance: L1-norm, weight: none, standardize: true, validation: tenfold CV.

*Cortex map generation:*

- Channel selection function: Median and accuracy-based selection,
- Creating: Intersection-based creation.

By deploying the above parameters, the proposed BWHPat-based EEG signal classification model has been created.

### Classification results

We evaluated our proposal using three generated cases, which are defined below:

*Case 1:* This case utilized EEG signals with a duration of 60 s. It consists of 109 low pain, 110 moderate pain, and 139 high pain EEG signals.

*Case 2:* The EEG signals used in this case are 30 s long. The distribution of observations across pain levels is: 219 low pain, 220 moderate pain, and 279 high pain.

*Case 3:* For this case, we used EEG signals of 15 s in duration. Out of the 1438 EEG observations, 439 are categorized as low pain, 440 as moderate pain, and 559 as high pain.

To assess the performance of our proposed model, we relied on two performance evaluation metrics: (i) classification accuracy and (ii) F1-score.

The mathematical definitions for these metrics are provided below (Powers 2020).

49 $$cac=\frac{tn+tp}{tn+tp+fn+fp}$$

50 $$F1=\frac{2tp}{2tp+fn+fp}$$

Herein, $tn,tp,fn$ and $fp$ are the number of true negatives, true positives, false negatives and false positives respectively, $F1$ defines the F1-score and F1-score is the harmonic mean of the recall and precision.

By using the proposed model, the computed performance metrics have been listed in Table 1.

**Table 1:** Results (%) of the presented BWHPat-based EEG pain classification model

No	Channel	Case 1 (60 s)		Case 2 (30 s)		Case 3 (15 s)	
		Acc	F1	Acc	F1	Acc	F1
1	Fp1	98.60	98.61	98.75	98.75	98.26	98.28
2	Fp2	98.04	97.99	98.89	98.88	98.68	98.67
3	AFz	**99.72**	**99.73**	99.58	99.59	**99.17**	**99.19**
4	F7	99.72	99.73	99.44	99.46	98.75	98.77
5	F3	98.04	98.09	99.03	99.02	98.89	98.91
6	Fz	98.32	98.33	97.91	97.96	98.19	98.24
7	F4	97.49	97.43	98.19	98.15	98.47	98.45
8	F8	98.60	98.61	98.89	98.92	98.96	98.97
9	T7	98.88	98.91	98.19	98.21	96.80	96.80
10	C3	97.77	97.82	97.91	97.93	98.05	98.06
11	Cz	98.60	98.60	98.75	98.73	98.12	98.13
12	C4	98.88	98.91	99.30	99.32	98.26	98.27
13	T8	98.32	98.33	97.91	97.95	97.98	97.99
14	CPz	99.44	99.46	99.30	99.32	98.33	98.33
15	P7	99.44	99.42	99.03	99.03	97.84	97.87
16	P3	99.16	99.18	98.89	98.88	98.54	98.55
17	Pz	98.88	98.88	98.47	98.47	99.17	99.17
18	P4	98.32	98.33	98.33	98.34	98.26	98.26
19	P8	98.60	98.53	97.77	97.79	97.71	97.72
20	POz	99.16	99.15	98.75	98.72	97.98	97.96
21	O1	99.16	99.18	98.75	98.78	97.71	97.73
22	O2	99.16	99.12	98.19	98.22	97.71	97.74
23	A1	99.16	99.18	98.47	98.46	98.47	98.46
24	A2	99.44	99.39	**99.72**	**99.73**	98.68	98.70

^**^ Acc.: Accuracy, F1: F1-score, the highest results have been highlighted using bold-font face

**Fig. 4:**
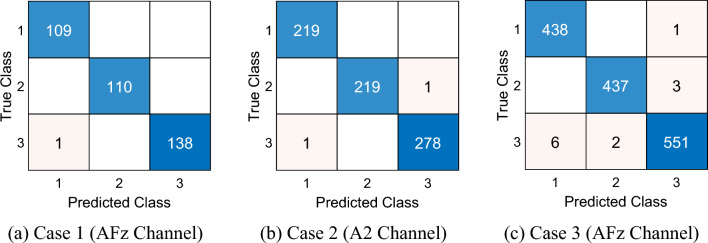
Confusion matrices of the best results of the cases. *1: low pain, 2: moderate pain, 3: high pain

Table 1 demonstrated that the proposed model attained 99.72%, 99.72% and 99.17% classification accuracies for Case 1, Case 2 and Case 3 consecutively. The best results have been attained using the AFz channel for Case 1 and Case 3 and the A2 channel for Case 2. The confusion matrices of the best results have also been illustrated in Fig. 4.

### Explainable results

**Fig. 5:**
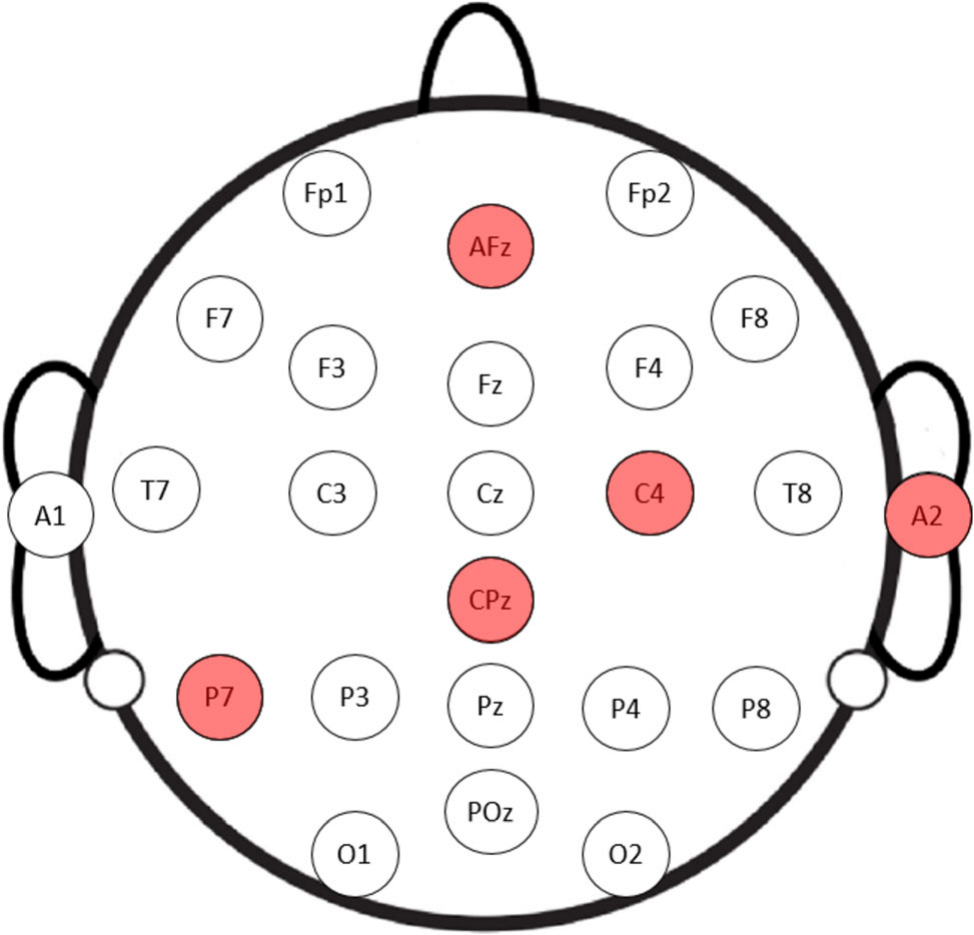
Schematic representation of most active channels used to classify chronic neuropathic pain

In the fourth phase of our model, we developed a semantic cortex map using a proposed median-based cortex map generator. The active channels for the three cases are illustrated in Fig. 5, based on the results of this intersection and median-based algorithm.

As shown in Fig. 5, the channels AFz, C4, CPz, P7, and A2 are the most distinguishable for chronic neuropathic pain classification.

The channels AFz, C4, CPz, P7, and A2 represent specific brain regions and functions relevant to the classification of chronic neuropathic pain based on EEG signals. AFz, associated with cognitive functions and attention, may reflect cognitive and attentional processes in pain perception. C4, responsible for motor control and somatosensory processing, can indicate changes in motor responses or sensory processing related to chronic neuropathic pain. CPz, involved in sensory integration and processing, may indicate disruptions in sensory processing linked to chronic neuropathic pain. P7, contributing to sensory and cognitive functions, including spatial awareness and attention, might reflect alterations in spatial perception or attention related to chronic neuropathic pain. A2, serving as a reference electrode, provides a stable reference point for EEG measurements during chronic neuropathic pain classification. Hence, these channels collectively play a pivotal role in chronic neuropathic pain classification, suggesting their involvement in various pain-related processes, sensory changes, and cognitive functions. Analyzing activity of these channels offer valuable insights into the neural mechanisms underlying chronic neuropathic pain.

## Discussions

**Fig. 6:**
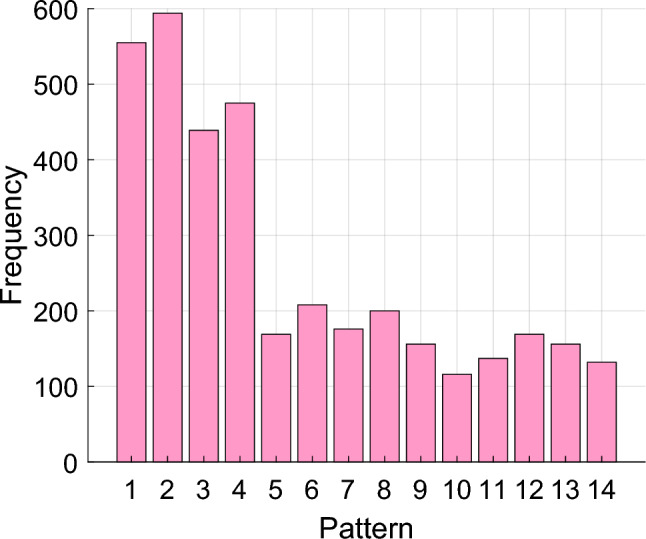
Histogram of the proposed BWHPat for a sample signal

Our work introduced a self-organized feature extraction function known as BWHPat which is helps to extract subtle patterns from the EEG signals. The histogram of the proposed BWHPat for various lengths of patterns are shown in Fig. 6.

Figure 6 indicates the ability of the BWHPat model to extract the minute features from the data. In Fig. 6, the second pattern emerges as the most frequently employed, underscoring the adaptability and effectiveness of BWHPat in the complex landscape of EEG signal analysis.

**Fig. 7:**
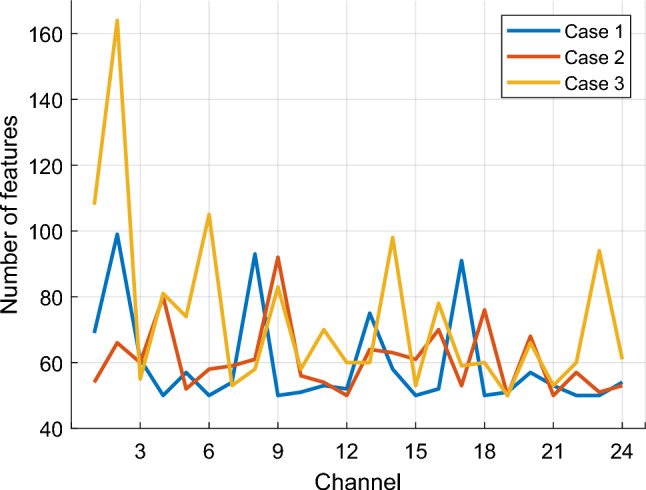
Number of selected feature vectors for different channels and cases

The INCA selector was employed during feature selection phase. This selector was applied for all 24 channels of our dataset. The count of features selected by the INCA selector for each case is shown in Fig. 7.

We employed the INCA selector for all 24 channels of our dataset. Figure 7 presents the count of features selected for each case, highlighting a range of feature lengths between 50 and 164. These results signify high classification performances and also emphasizes the ability to obtain high performance with few selected features.

**Fig. 8:**
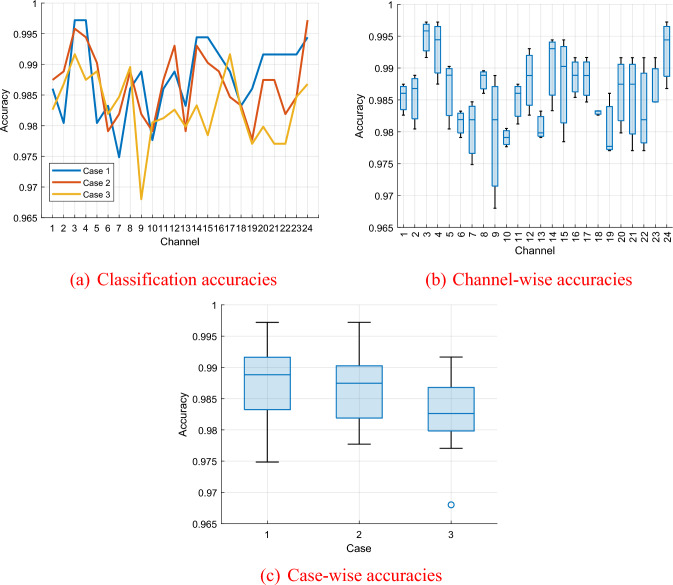
Summary of classification accuracies obtained. **a** Classification accuracies, **b** Channel-wise accuracies, and **c** Case-wise accuracies

Moreover, we compared the classification performances across the three cases. The classification accuracies of the proposed BWHPat-based EEG signal classification model for each case, along with their statistical illustrations, are presented in Fig. 8.

Figure 8(a) indicate the computed classification accuracies of the developed model. Channel-wise analysis in Fig. 8(b) indicate that the 3rd channel (AFz) is the best performing one yielding an accuracy of 99.49% ± 0.29% accuracy, while the 10th channel (C3) obtained a lower accuracy of 97.91% ± 0.14%. The classification performances in Fig. 8(c) further underscores the closely matched successes of Case 1, Case 2, and Case 3.

A semantic cortex map showcased in Fig. 5, offering a visual narrative of the profound effects of chronic neuropathic pains. The visual revelation emphasizes the frontal, central, and parietal areas as primary targets, providing critical insights into the neurological repercussions of chronic pain.

In the neuroanatomy of pain perception, our study helps to understand the peripheral pain signals through the spinal cord to the somatosensory cortex. The interconnected brain regions, including the somatosensory cortex, periaqueductal gray matter, amygdala, hypothalamus, ventral tegmental area, and nucleus accumbens, unfold as central players in the supraspinal pain processing saga. The researchers Yang and Chang (Yang and Chang 2019) indicated that the anterior-central nucleus accumbens and the posterior-central ventral tegmental areas are the key players in chronic pain perception.

Our EEG data analysis indicated perturbations in channels AFz, C4, CPz, and P7, strategically aligning with regions crucial to pain processing within the parietal cortex. This not only substantiates our findings but also strengthens the argument for the effectiveness of our model in capturing important features and classification of neurological abnormalities.

Hence, BWHPat is a feature extraction function and also EEG signal classification model.

### Ablations

In this section, we present ablation results to highlight the superior classification performance of the proposed BWHPat-based model. For these results, we used the AFz channels. Three distinct configurations were set up to demonstrate the effectiveness of the introduced BWHPat. These configurations are detailed below:

*Item 1:* This configuration employs the local binary pattern (LBP) as our architecture's primary feature extraction function.

*Item 2:* Here, the proposed BWHPat serves as the main feature extraction function.

*Item 3:* Both BWHPat and statistical feature extractors are utilized for feature extraction.

**Fig. 9:**
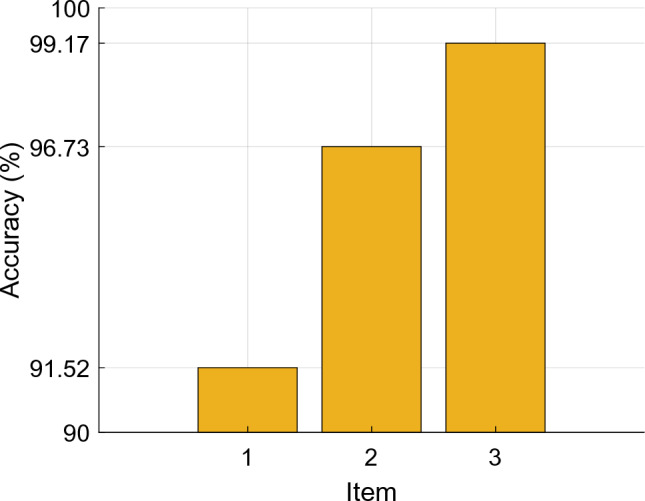
Classification accuracies obtained for different items

Figure 9 shows that our proposed BWHPat outperforms the LBP, with our feature extractor (BWHPat) achieving a classification accuracy that is 5.21% higher than that of LBP. By integrating our approach (BWHPat combined with statistical features), we achieved a classification accuracy of 99.17%. The results in Fig. 9 unequivocally show that our chosen strategy offers the best combination.

### Highlights

The findings and advantages of the proposed model are given below.

*Findings:*

1. Efficiency of BWHPat feature extractor:
  - The proposed BWHPat function exhibits highly effective self-organized feature extraction, adapting dynamically to data blocks and selecting patterns tailored to the characteristics of the input.
  - Proposed BWHPat performed better than LBP, achieving a classification accuracy of 5.21% higher.
2. Robust performance and adaptability:
  - Integration of BWHPat with statistical features results in a robust classification accuracy of 99.17%, emphasizing its adaptability and effectiveness, particularly for the AFz channel in Case 3.
  - The model demonstrates versatility by achieving optimal classification performance across different cases, using fewer features (ranging from 50 to 164) for effective signal analysis.
3. Channel-wise analysis:
  - The channel-wise analysis identifies the 3rd channel (AFz) as the most efficient, attaining a mean classification accuracy of 99.49% ± 0.29%, while the 10th channel (C3) shows relatively lower accuracy at 97.91% ± 0.14%.
  - Notably, the model maintains high classification accuracies across all channels, highlighting its consistency in performance.
4. Case-wise analysis:
  - Overall classification accuracies for Case 1, Case 2, and Case 3 are 98.79% ± 0.60%, 98.68% ± 0.56%, and 98.29% ± 0.57%, respectively. Case 1 exhibits the best performance, although performances across cases are closely matched.
  - Specific channels (AFz, A2, and AFz) contribute to the best classification accuracies for each case, emphasizing the significance of channel selection in achieving optimal results.
5. Semantic cortex map generation:
  - The generated semantic cortex map reveals that chronic neuropathic pains predominantly impact the frontal, central, and parietal lobes of the brain.
  - This visualization provides valuable insights into the specific brain regions affected by chronic neuropathic pains, contributing to a better understanding of the neurological implications.

The advantages in two categories: (i) technical and (ii) practical are given below.

*Technical advantages:*

- The BWHPat can self-organize and select the most appropriate pattern from the data block.
- The model performed better than the traditional LBP in terms of feature extraction.
- To the best of our knowledge, we are the first team to propose an astronomy-related feature extraction method. for the main contribution of this work is the astronomy-driven feature extraction methodology for neuroscience.
- Fusion of BWHPat with statistical features resulted in high classification accuracy.
- The model effectively handles various features, ranging from 50 to 164, to achieve optimal performance.
- The model's design facilitates a channel-wise accuracy analysis, helping to pinpoint the most and least efficient channels.
- The semantic cortex map provides a comprehensive view of the regions affected by chronic neuropathic pains.
- The model's integration of multiple methods (like TQWT, statistical feature extractor, INCA, kNN, etc.) provides a robust and comprehensive approach to EEG signal classification.

*Practical advantages:*

- The reported high classification accuracy in the paper has substantial implications for clinical and practical applications. The generated cortex map, significantly contributes to our understanding of pain-related brain activity, opening avenues for practical implementation and clinical relevance.
- The generated cortex map visually illustrates the regions predominantly affected by chronic neuropathic pains, specifically highlighting the frontal, central, and parietal lobes. This information is invaluable in clinical contexts as it allows for more precise localization of pain-related brain activities. Clinicians can utilize this data to target interventions to the specific areas of the brain implicated in pain perception, potentially improving the precision of treatments.
- The high classification accuracy achieved by the proposed model suggests its application as a diagnostic tool in clinical settings. Accurate classification of EEG signals related to neuropathic pain can aid healthcare practitioners in effective diagnosis of pain. This can help in early interventions and personalized treatment plans tailored to the individual's neural responses.
- The ability to generate a cortex map based on EEG signals provides an objective and quantifiable measure of pain-related brain activity. This can help to assess the pain intensity objectively, reducing reliance on subjective self-reports. Healthcare providers can use the generated cortex map to obtain an objective metric of pain perception, facilitating more accurate pain management.
- The high classification accuracy of the proposed model suggests its potential application in monitoring the efficacy of pain management strategies over time. By observing changes in the cortex map patterns, clinicians can assess the impact of therapeutic interventions and make decisions to optimize treatment plans based on observed neural responses. This contributes to a more dynamic and personalized approach to pain management.
- In addition to the clinical applications, this study contributes to advancing our broader understanding of pain-related brain activity. The observed perturbations in specific channels aligning with regions implicated in pain processing provide valuable insights for neuroscientific research. This knowledge contributes to the ongoing discourse on the neural mechanisms underlying chronic neuropathic pains.

The high classification accuracy and the generation of a cortex map have practical implications in precise pain localization, enhanced diagnostics, objective pain assessment, treatment monitoring, and advancements in neuroscientific research. These findings pave the way for improved clinical practices and a deeper understanding of the neural correlates of chronic neuropathic pains.

### Limitations and future works

**Fig. 10:**
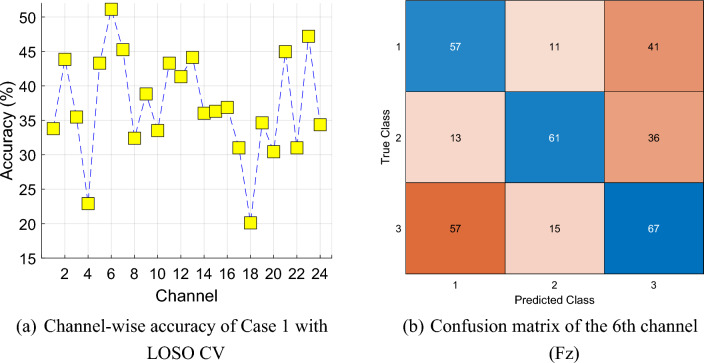
LOSO CV-based results

This research used a publicly available dataset collected from 36 participants. We applied the leave-one subject-out (LOSO) cross-validation and attained about 51% classification accuracy. We have tested this dataset using LOSO CV and the classification accuracies of all channels and confusion matrix of the best-resulted channel have been illustrated in Fig. 10.

The LOSO CV results demonstrated that (see Fig. 10) a bigger dataset can be collected and a healthy control group can be added. Moreover, our future intention is to propose more astronomy-related self-organized feature extraction functions.

The limitations and future works are also discussed below.

*Limitations:*

- The presented work was developed using a public database consisting of 36 participants. The limited size of the dataset might impact the generalizability of the findings.
- about the developed model yielded only 51% classification accuracy with LOSO cross-validation strategy, which demands the need for the huge dataset.

*Future directions:*

- Huge dataset with a control group helps to develop a robust model.
- The clinical application of the model need to be validated in real-world scenarios, considering factors such as patient heterogeneity, different pain types, noise, and comorbidities. Uncertainty quantification technique may help to overcome the problem due to noise in the model (Seoni et al. 2023).
- Further exploration into astronomy-related self-organized feature extraction functions can provide unique insights and contribution to the neuroscience field.
- Combining EEG data with other neuroimaging modalities or clinical parameters can offer a more comprehensive understanding of pain-related brain activity (Salvi et al. 2023).
- Conducting longitudinal studies would help in capturing the dynamic nature of neuropathic pain, enabling the model to adapt to changes over time and providing more accurate predictions.

Addressing these limitations and exploring these future TQWT is chosen directions can contribute to the EEG-based pain classification model in clinical settings and pave the way for advancements in pain-related brain activity research.

## Conclusions

In our research, we have developed a novel self-organized feature extraction function, BWHPat, tailored for EEG signal classification related to neuropathic pain. The incorporation of this function within our model, which also integrates state-of-the-art techniques like TQWT, INCA, kNN, and a distinctive cortex map generator, signifies a comprehensive approach to EEG signal analysis.

From our empirical findings:

- The proposed BWHPat demonstrated a clear edge over traditional methods such as LBP, with an improvement of over 5.21% classification accuracy.
- Channels like AFz exhibited remarkable classification accuracy, reaching up to 99.49% ± 0.29%.
- When examining different cases based on signal lengths, our model consistently maintained high classification accuracies, with Case 1, Case 2, and Case 3 achieving 98.79% ± 0.60%, 98.68% ± 0.56%, and 98.29% ± 0.57%, respectively.
- The combined strategy of BWHPat and statistical features reached impressive classification accuracies. The best classification accuracies for Case 1, Case 2 and Case 3 were computed as 99.72%, 99.72% and 99.17% respectively.

Our generated semantic cortex map offers a new perspective on the effects of chronic neuropathic pain across various brain regions. These numerical outcomes underscore the model's robust performance and its capability to deliver consistently across different EEG signal durations.

The proposed BWHPat-based model significantly advances EEG analysis for neuropathic pain classification. It combines high classification accuracy with rich information, creating a reference point for future works in this area.

## Data Availability

The used dataset can be downloaded using (Zolezzi et al. 2023a, 2023b).
